# MicroRNA-132 promotes estradiol synthesis in ovarian granulosa cells via translational repression of *Nurr1*

**DOI:** 10.1186/s12958-015-0095-z

**Published:** 2015-08-19

**Authors:** Shaogen Wu, Haixiang Sun, Qun Zhang, Yue Jiang, Ting Fang, Isabelle Cui, Guijun Yan, Yali Hu

**Affiliations:** Reproductive Medicine Center, Department of Obstetrics and Gynecology, Nanjing Drum Tower Hospital, Nanjing University Medical School, Nanjing, China; New York-Presbyterian/Weill Cornell Medical Center, New York, NY USA

**Keywords:** microRNA-132, Estradiol, Granulosa cell, Nurr1, Cyp19a1

## Abstract

**Background:**

Estrogen synthesis is an important function of the mammalian ovary. Estrogen plays important roles in many biological processes, including follicular development, oocyte maturation and endometrial proliferation, and dysfunctions in estrogen synthesis contribute to the development of polycystic ovary syndrome and premature ovarian failure. Classical signaling cascades triggered by follicle-stimulating hormone induce estrogen synthesis via the upregulation of *Cyp19a1* in granulosa cells (GCs). This study aimed to determine the effect of microRNA-132 (miR-132) on estradiol synthesis in GCs.

**Methods:**

Primary mouse GCs were collected from ovaries of 21-day-old immature ICR mice through follicle puncture. GCs were cultured and treated with the stable cyclic adenosine monophosphate analog 8-Br-cAMP or transfected with miR-132 mimics, *Nurr1*-specific small interfering RNA oligonucleotides and Flag-Nurr1 plasmids. Concentrations of estradiol and progesterone in culture medium were determined by an automated chemiluminescence-based assay. Quantitative real time PCR and western blot were performed to identify the effect of miR-132 on *Cyp19a1*, *Cyp11a1* and an orphan nuclear receptor-Nurr1 expression in GCs. Direct suppression of *Nurr1* via its 3'-untranslated region by miR-132 were further verified using luciferase reporter assays.

**Results:**

The expression level of miR-132 in cultured mouse GCs was significantly elevated during 48 h of treatment with 8-Br-cAMP. The synthesis of estradiol increased after the overexpression of miR-132 in mouse GCs. The real-time PCR results demonstrated that miR-132 induced the expression of *Cyp19a1* significantly. Nurr1, an orphan nuclear receptor that suppresses *Cyp19a1* expression, was found to be a direct target of miR-132. *Nurr1* was suppressed by miR-132*,* as indicated by a luciferase assay and Western blotting. The knockdown of *Nurr1* primarily elevated the synthesis of estradiol and partially attenuated the miR-132-induced estradiol elevation, and the ectopic expression of Flag-Nurr1 abrogated the stimulatory effect of miR-132 on estradiol synthesis in mouse GCs.

**Conclusions:**

Our findings suggest that miR-132 is involved in the cAMP signaling pathway and promotes estradiol synthesis via the translational repression of *Nurr1* in ovarian GCs.

## Background

Ovarian steroid hormones such as estradiol (E_2_) play important roles in many biological processes, including ovarian follicular development, oocyte maturation, endometrial proliferation and mammary gland development [1, 2]. In addition, dysfunctions in estrogen synthesis are associated with the development of polycystic ovary syndrome and premature ovarian failure [3, 4]. According to the traditional two-step theory of E_2_ biosynthesis, androgen is produced from cholesterol in theca cells and converted into E_2_ via cytochrome P450 aromatase, a rate-limiting enzyme for estrogen synthesis, in granulosa cells (GCs) [5]. Follicle-stimulating hormone (FSH) is a glycoprotein hormone that is produced by the anterior pituitary gland. This gonadotropin plays an essential role in steroidogenesis of ovarian GCs. The binding of FSH to its receptor (FSHR) on the surface of GCs in immature preantral follicles activates the effector adenylyl cyclase, which leads to the synthesis and upregulation of the intracellular second messenger cyclic adenosine monophosphate (cAMP) [6]. By activating multiple signaling cascades, FSH triggers the specific, time-related expression of genes, such as *Cyp19a1*, and promotes the proliferation and differentiation of GCs [7]. FSH induces the phosphorylation of the cAMP response element binding protein (CREB), which transactivates *Cyp19a1* by binding to a cAMP-responsive element-like sequence (CLS) in its proximal promoter (PII promoter) [8–10]. Besides classical regulations in the FSH pathway, epigenetic mechanisms remain to be elucidated, which will increase our understanding of ovarian physiology.

MicroRNAs (miRNAs) are small noncoding RNAs that are 20-24 nucleotides in length and are endogenously expressed in most eukaryotes. Previous studies demonstrated that miRNAs play important roles in diverse biological processes, such as development, inflammation and tumorigenesis [11]. The primary mechanism by which miRNAs regulate gene expression is via posttranscriptional binding to the 3'-untranslated region (3'-UTR) of mRNAs, which leads to either degradation or translational repression of the mRNA. In the ovary, many miRNAs are involved in the proliferation, apoptosis, and differentiation of GCs [12, 13]. Some miRNAs have recently been reported to influence steroid hormone release from human ovarian GCs based on a genome-scale miRNA screen [14]. Studies examining miRNA-regulated E_2_ biosynthesis determined that miR-224 [15] and miR-383 [16] play important roles in the TGF-β/Smads pathway by targeting *Smad4* and *RBMS1*, respectively. The Cyp19a1 gene has also been confirmed to be a direct target of miR-378 [17] and miR-98 [18].

Among the miRNAs that are involved in the cAMP signaling pathway, miR-132 has been demonstrated to be upregulated in rat GCs by either cAMP [19] or FSH treatment [20] and in periovulatory mouse granulosa cells (mGCs) after LH/hCG induction [21]. A recent study in polycystic ovary syndrome patients showed that the expression levels of miR-132 in follicular fluid were significantly lower in patients than in controls [22]. They also found that overexpression of miR-132 increased E_2_ secretion from KGN, a steroidogenic human granulosa-like tumor cell line. These findings suggest that miR-132 may play diverse roles such as steroidogenesis in different developmental stage of granulose cells. The functions of miR-132 may be related to the fact that cAMP mediates divergent pathways depending on the differential status of GCs [23]. Our aims of this study are to determine if miR-132 is involved in the cAMP pathway in primary cultured mGCs isolated from immature mice and to investigate the role of miR-132 in E_2_ synthesis using a relatively low plating density to retain the estrogenic phenotype of mGCs [24]. Our study also identified *Nurr1* as a direct target of miR-132, which mediates the regulation of E_2_ synthesis by miR-132 in mGCs.

## Methods

### Animals

Three-week-old ICR mice were purchased from the Lab Animal Center of Yangzhou University (Yangzhou, China). All animals were maintained in the Animal Laboratory Center of Drum Tower Hospital (Nanjing, China) on a 12-h/12-h light/dark cycle (lights off at 19:00), with food and water available *ad libitum*. All animal experiments were approved by the Institutional Animal Care and Use Committee at Nanjing Drum Tower Hospital (SYXK 20014-0052).

### Isolation and culture of primary mGCs

A previously described in-house method [25] was performed to isolate mGCs from the ovaries of 21-day-old immature mice. Briefly, the ovaries were harvested and separated from the surrounding fat. After the ovaries had been punctured repeatedly with 25 gauge needles, the mGCs were collected and plated in DMEM/F12 (Gibco, Life Technologies, Carlsbad, CA, USA) containing 10 % FBS (Gibco), 1 mM sodium pyruvate (HyClone, Thermo Scientific, South Logan, UT, USA), 2 mM L-glutamine (Gibco), and 1 % antibiotics (100 U/ml penicillin and 100 μg/ml streptomycin; Gibco). The medium was replaced 24 h after plating to remove any unattached cells. The mGCs were cultured in medium at 37 °C in a humidified environment with 5 % CO_2_ and were used after the first passage. At 24 h after plating, the cells were placed in phenol red-free DMEM/F12 (HyClone) supplemented with 2 % charcoal/dextran-treated fetal bovine serum (C-FBS; HyClone) for 48 h. The cells were subsequently treated with medium alone or with 1 mM 8-bromoadenosine 3',5'-cyclic monophosphate (8-Br-cAMP) (Sigma, St. Louis, MO, USA) for 0, 3, 6, 12, 24 or 48 h. Total RNA was isolated, and the expression of miR-132 was analyzed using quantitative polymerase chain reaction (PCR).

### Immunofluorescence staining

mGCs were plated on 18 mm microcover glasses (Matsunami, Osaka, Japan) for 24 h and subsequently fixed with 4 % paraformaldehyde in PBS for 30 min at room temperature. The cells were then washed with PBS and permeabilized with 0.2 % Triton X-100 in PBS for 15 min at room temperature. After being blocked with 1 % BSA in PBS, cells were stained for FSHR by incubation with a 1:100 dilution of an anti-FSHR polyclonal antibody (Bioworld Technology, St. Louis Park, MN, USA), followed by incubation with a 1:200 dilution of an Alexa Fluor 488-conjugated goat anti-rabbit IgG (Molecular Probes, Life Technologies, Carlsbad, CA, USA) for 1 h. PBS was used as negative controls for primary and secondary antibodies to exclude nonspecific staining. Nuclei were stained with a 1:5000 dilution of DAPI (Vector Laboratories, Burlingame, CA, USA). Images were visualized using a FLUOVIEW FV10i confocal microscope system (Olympus, Tokyo, Japan).

### Immunohistochemistry

Formalin-fixed paraffin-embedded 21-day-old immature mice ovaries were serially sectioned, dewaxed with xylene and rehydrated through a graded alcohol series. Sections were then treated with 3 % hydrogen peroxide to quench endogenous peroxidase activity, microwaved sequentially to retrieve antigen, and incubated in blocking solution for 1 h. Sections were then incubated with a 1:100 dilution of an anti-FSHR polyclonal antibody (Bioworld Technology) overnight at 4 °C. The next day, the sections were incubated with goat anti-rabbit secondary antibody ABC detect kit (ZSBio, Beijing, China) at 37 °C for 30 min, and then stained with 3,30-diaminobenzidine (DAB) and counterstained with hematoxylin. Negative control sections were processed concurrently using PBS and similarly pre-treated.

### Plasmid construction

*NURR1* cDNA [GeneBank: NM_006186.3] was synthesized and amplified from the total RNA of human endometrial stromal cells using the SuperScript III One-Step RT-PCR System with the Platinum Taq High Fidelity Kit (Invitrogen, Life Technologies, Carlsbad, CA, USA) and the following primers: 5'-CGACACTGTCCACCTTTAATTTC-3' and 3'-TTTAGGGATCAAGGGGGCTA-5'. A second PCR step was performed using the Platinum Pfx DNA Polymerase (Invitrogen) and the following primers: 5'-TATAAGATCTGATGCCTTGTGTTCAGGCGCAG-3' and 5'-TAGCGGTACCTTAGAAAGGTAAAGTGTCCAG-3'. To create a Flag-Nurr1 protein expression vector, fragments harboring full-length *NURR1* were cloned into pFLAG-CMV-2 (Sigma) using the BglII and KpnI restriction sites (Promega, Madison, WI, USA). The wild-type sequence of the Nurr1 3'-UTR [GeneBank: NM_013613.2] that contains the miR-132 binding site was amplified using mGC cDNA as a template and the following primers: 5'-TATCTCGAGGAATTGAAGGCAGAGGCTTG-3' and 5'-TCGTCTAGATGACTCATCTCATGTGCCGTA-3'. To create the pmirGLO-Luc-Nurr1 3'-UTR WT vector, the resulting PCR fragment was cloned into the pmirGLO dual-luciferase miRNA target expression vector (Promega) using the XhoI and XbaI restriction sites (Promega). The mutant sequence contained two mutations in the ‘seed sequence’ of the miR-132 binding site, which is indicated in Fig. 5a. We designed primers (5'-CAGCTTTTGGATGTTTCCAGAG-3' and 5'-CACTCTGGAAACATCCAAAAGC-3') to create a pmirGLO-Luc-Nurr1 3'-UTR MU vector via overlap extension PCR. A luciferase reporter gene plasmid containing NGFI-B response elements upstream of the reporter (NBRE-Luc) was constructed according to previously described methods [26] using the pGL3-Basic vector (Promega), which was a generous gift from Sun Jianxin at Thomas Jefferson University, Philadelphia, USA. The sequences of all recombinant plasmids were confirmed by DNA sequencing.

### Transient transfection

Chemically synthesized single-stranded RNAs that mimic mature endogenous miR-132 [GeneBank: NR_029546.1] after transfection into cells were used as miR-132 mimics, and mimics NC were used as negative controls. Chemically modified antisense RNA oligonucleotides optimized to specifically target miRNA molecules in cells were used as miRNA inhibitors, and inhibitors NC were used as negative controls. Nurr1-specific siRNA oligonucleotides (sense: 5'-CCACCUUGCUUGUACCAAAdTdT-3'; antisense: 3'-dTdT GGUGGAACGAACAUGGUUU-5') were used to knock down endogenous Nurr1, and siNC oligonucleotides were used as negative controls. These oligonucleotides were purchased from Ribobio (Guangzhou, China). Primary mGCs were transfected with either oligonucleotides or plasmids using Lipofectamine 2000 (Invitrogen) according to the manufacturer’s protocol. For each transfection, a final oligonucleotide concentration of 100 nM was used.

### Western blot analysis

Total protein was isolated from mGCs that were harvested 48 h after treatment. The cells were rinsed twice with ice-cold PBS (pH 7.4) and lysed with whole lysis buffer (50 mM Tris-HCl, pH 7.6; 150 mM NaCl; and 1.0 % NP-40) containing protease inhibitor cocktail (Sigma). The protein concentrations were measured using the Pierce BCA protein assay (Thermo). Equal amounts of total protein (40 μg) were separated on a 10 % SDS-polyacrylamide gel and transferred to a polyvinylidene fluoride membrane (Millipore, Billerica, MA, USA). Immunoblotting was performed using primary antibodies against Nurr1 (1:1000; R&D Systems, Minneapolis MN, USA) and Nur77 (1:500; Santa Cruz, CA, USA). Glyceraldehyde-3-phosphate dehydrogenase (GAPDH) was selected as an internal control and was visualized using rabbit anti-GAPDH IgG (1:10000; Bioworld). Immunodetection was accomplished using a goat anti-rabbit IgG (1:5000; GenScript, Piscataway, NJ, USA) or a donkey anti-goat IgG (1:5000; Santa Cruz) secondary antibody and an enhanced chemiluminescence detection kit (Millipore) with the Clinx Chemiscope 3400 Mini Western Blot Imaging System (Clinx Science Instruments, Shanghai, China). Signals from the Western blot images were quantified by measuring the optical density of each band. The blot density of the control was set as 100 %. After normalization to the corresponding GAPDH band, the relative density values of other bands were calculated by dividing the optical density values by the control value. All experiments were repeated three times.

### RNA extraction and quantitative real-time PCR

Total RNA was extracted from cultured cells using the TRIzol reagent (Invitrogen). cDNA was synthesized from 1 μg of purified total RNA using the PrimeScript RT Reagent Kit with gDNA Eraser (Takara, Dalian, China) according to the manufacturer’s instructions with either the random primers provided in the kit or specific reverse primers (miR-132: 5'-CTCAACTGGTGTCGTGGAGTCGGCAATTCAGTTGAGCGACCATG-3', U6: 5'-AACGCTTCACGAATTTGCGT-3'). The specific primers used for real-time PCR analysis are listed in Table 1. Each 20 μL real-time PCR reaction had the following components: 2 μL of RT product (equivalent to 20 ng of total RNA), 10 μL of iQ SYBR Green Supermix (Bio-Rad Laboratories, Hercules, CA, USA), and 250 nM forward and reverse primers. Real-time PCR for gene transcription was performed on a MyiQ Single Color Real-time PCR Detection System (Bio-Rad Laboratories). The cycle parameters for miRNAs were as follows: an initial 15 min incubation at 95 °C, followed by 40 cycles of 95 °C for 15 s and 60 °C for 1 min. The cycle parameters for genes were as follows: an initial 3 min incubation at 95 °C, followed by 40 cycles of 95 °C for 10 s, 60 °C for 30 s, and 72 °C for 30 s. The data were analyzed using the 2^-ΔΔCt^ method [27], and the obtained fold changes in miRNA or gene expression were normalized to U6 snRNA or 18S rRNA as endogenous controls, respectively. Each sample was analyzed in triplicate, and the experiments were repeated three times.

**Table 1:** Sequences of primers used for real-time PCR analysis

Gene	Forward primer (5' → 3')	Reverse primer (5' → 3')
miR-132	ACACTCCAGCTGGGTAACAGTCTACAGCCA	GGTGTCGTGGAGTCGGCAATTCAGTTGAG
U6 snRNA	CTCGCTTCGGCAGCACA	AACGCTTCACGAATTTGCGT
*Cyp19a1*	TGTGTTGACCCTCATGAGACA	CTTGACGGATCGTTCATACTTTC
*Cyp11a1*	TCCCTGTAAATGGGGCCATAC	AGGTCCTTCAATGAGATCCCTT
*Nurr1*	GATCGAGCAGAGGAAGAC	AAGCGCATCTGGCAGCTA
18S rRNA	ATGGCCGTTCTTAGTTGGTG	CGGACATCTAAGGGCATCAC

### Luciferase reporter assay

mGCs with a confluency of ~60 % were transfected with luciferase reporter plasmids and miR-132 mimics/inhibitors or the corresponding negative controls. All cells were co-transfected with the *Renilla* luciferase reporter plasmid (pRL-RSV; Promega) as a control for transfection efficiency. Luciferase activity was assayed 48 h after transfection using the Dual-Luciferase Reporter Assay System (Promega), and the ratio of firefly luciferase to *Renilla* luciferase was measured using a Centro XS3 LB 960 Microplate Luminometer (Berthold Technologies, Bad Wildbad, Germany). At least three transfection assays were performed to obtain statistically significant data.

### Hormone assays

For hormone assays, mGCs were cultured in 12-well plates in phenol red-free DMEM/F12 (Hyclone) supplemented with 2 % C-FBS (HyClone) and 2 μM 4-androstene-3, 17-dione (Sigma). To determine the effects of 8-Br-cAMP on mGC function, mGCs were treated with medium alone or with 1 mM 8-Br-cAMP (Sigma) for 24 h or 48 h. To determine the effects of miR-132 on mGCs, the medium was changed 6 h after transfection with miR-132 mimics/inhibitors or the corresponding negative controls, and the cells were cultured for an additional 48 h. To determine the effect of Nurr1 on mGCs, siNurr1 was transfected into cells 24 h prior to the transfection of miR-132 mimics, and the cells were cultured for an additional 24 h or 48 h. Culture medium was collected at the indicated time points, and the concentrations of E_2_ and progesterone in the culture medium were determined using the Access Immunoassay System 2 (Beckman Coulter, Brea, CA, Germany), an automated random-access chemiluminescence-based assay. The intra- and interassay coefficients of variation were less than 10 % and 15 %, respectively. Each assay was performed in triplicate, and the experiments were repeated at least three times.

### Statistical analysis

Data were expressed as the mean +/- SEM of at least three independent experiments. Student’s *t*-test was performed for comparisons of the mean values of two groups; one-way ANOVA was used to determine differences among the mean values of more than two groups because the quantitative data followed a normal distribution. *P* values less than 0.05 were considered statistically significant.

## Results

### miR-132 expression is responsive to 8-Br-cAMP stimulation

**Fig. 1:**
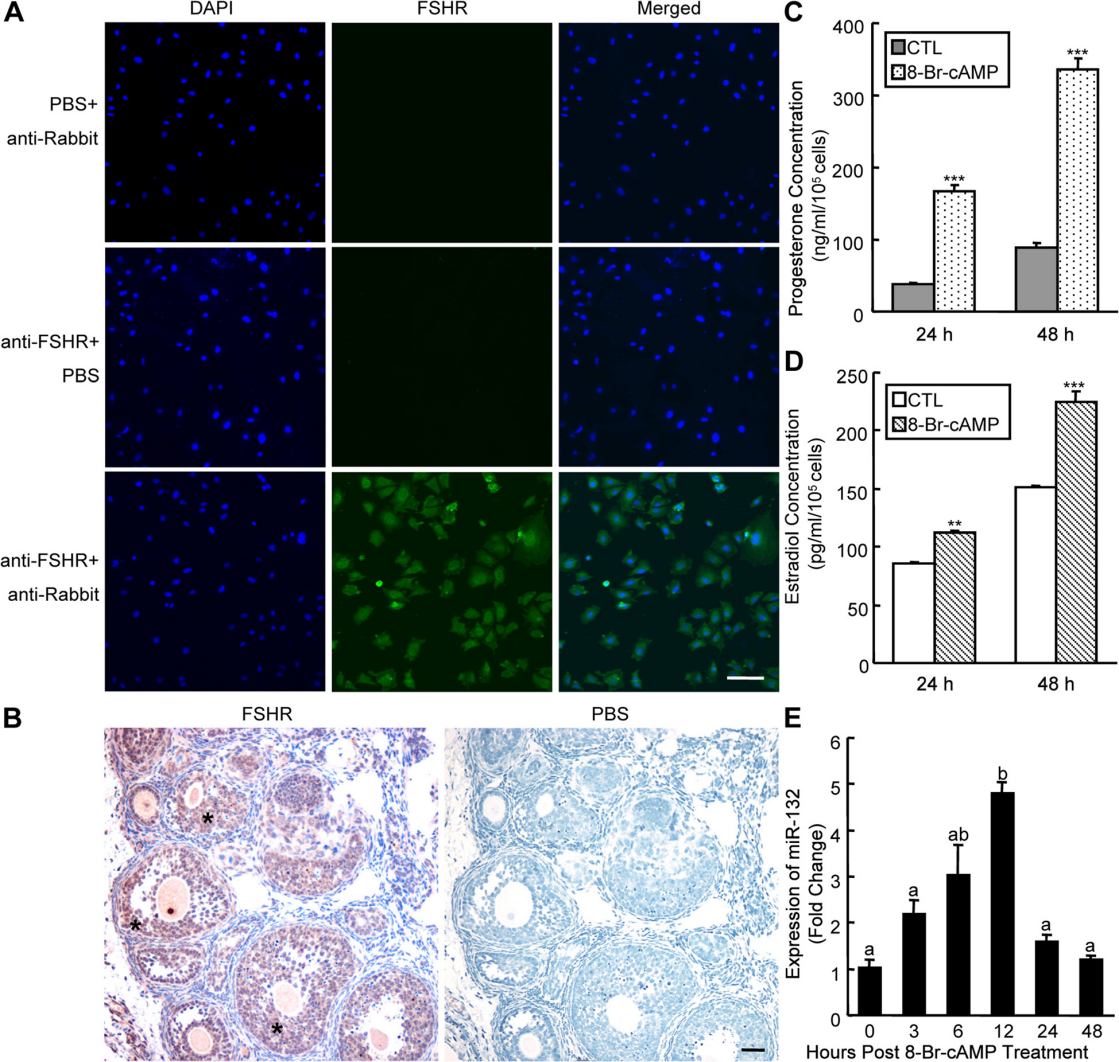
8-Br-cAMP treatment increases miR-132 expression and steroidogenesis in mGCs. **a** Primary mGCs were isolated from 21-day-old mouse ovaries. FSHR protein of mGC was detected by immunofluorescence staining (FSHR: green, DAPI: blue). Nonspecific staining was visualized using PBS to replace either the primary or secondary antibodies. **b** Immunohistochemical detection of mGCs (indicated by asterisks) in 21-day-old mouse ovaries. Immunostaining of FSHR is shown in brown, and nonspecific staining was visualized using PBS. Hematoxylin nuclear staining is shown in blue. The concentrations of progesterone (**c**) and E_2_ (**d**) per 10^5^ cells in culture medium were determined after 8-Br-cAMP treatment for 24 h and 48 h. **e** At the indicated time points after treatment with 8-Br-cAMP, the miR-132 levels in mGCs were determined using qRT-PCR. The results were normalized to U6 as an internal control. The results represent the mean +/- SEM of three independent experiments. Values with different superscripts (a, b) are significantly different (*p* < 0.05). **p < 0.01; ***p < 0.001, compared with the control (CTL). Scale bar: 100 μm

As shown in Fig. 1a, we successfully established monolayers of mGCs from the ovaries of 21-day-old immature mice. Most of the cells in the cultures were mGCs, which were characterized by positive FSHR staining. FSHR expression also showed that our *in vitro* cultured primary mGCs can maintain an estrogenic stage and can be used for further study of E_2_ synthesis. To test the specificity of anti-FSHR antibody, we did immunohistochemistry to show that our primary anti-FSHR antibody exclusively detected FSHR (stained brown) mainly in the mGCs at various stages of follicular development. Non-specific staining was not detected with PBS (Fig. 1b). The secretion of progesterone and E_2_ by cultured mGCs was significantly increased (4.6- and 1.3-fold, respectively) after exposure to 8-Br-cAMP, a stable cell-permeable analog of cAMP, for 24 h (Fig. 1c, d). The stimulatory effect of 8-Br-cAMP on E_2_ reached a greater extent (1.5-fold) after continuous treatment with 8-Br-cAMP for 48 h (Fig. 1d). cAMP is the crucial second messenger that is downstream of FSH in the FSH-mediated ovarian GC differentiation pathway. At least four cAMP-response element (CRE) sites are involved in miR-132 transcription in mice [28]. Previous studies have demonstrated that miR-132 levels are elevated in periovulatory mGCs and upregulated by hCG/LH, cAMP and FSH [19–21]. To determine whether miR-132 is induced by the cAMP signal transduction pathway, primary mGCs were exposed to 8-Br-cAMP for 0 to 48 h. During this period, miR-132 expression was continuously elevated, peaking at 12 h (~5-fold increased) and dropped to basal level after 24 h (Fig. 1e). The observed pattern of miR-132 upregulation is consistent with reported increases in steroid hormone release from mGCs, suggesting that miR-132 is involved in cAMP-mediated pathways, such as those that are important for the differentiation of GCs.

### miR-132 enhances the synthesis of E_2_ in mGCs

**Fig. 2:**
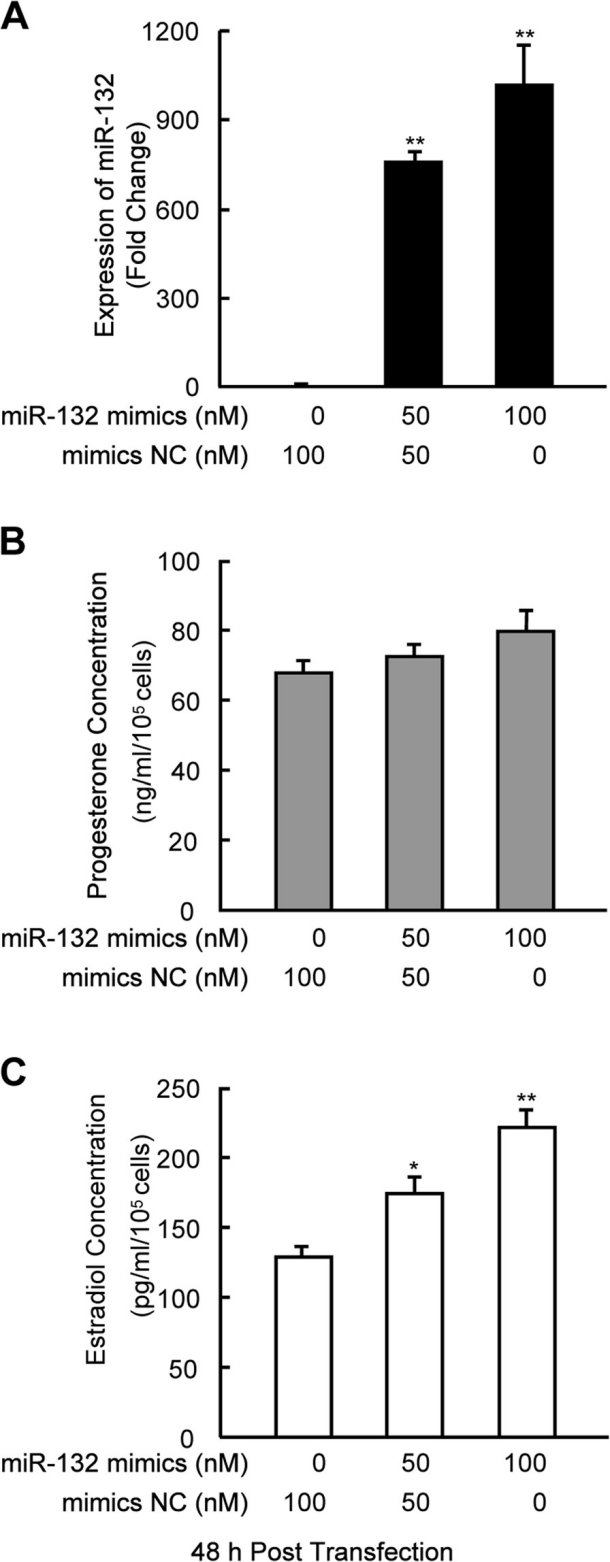
Overexpression of miR-132 enhances E2 synthesis in mGCs. **a** mGCs were transfected with miR-132 mimics or negative controls at the indicated concentration. The miR-132 levels were detected by qRT-PCR 48 h after transient transfection. The culture medium was collected for the measurement of progesterone (**b**) and E_2_ (**c**) per 10^5^ mGCs. The results represent the mean +/-SEM of three independent experiments performed in triplicate. *p < 0.05; **p < 0.01, compared with the negative control (NC)

**Fig. 3:**
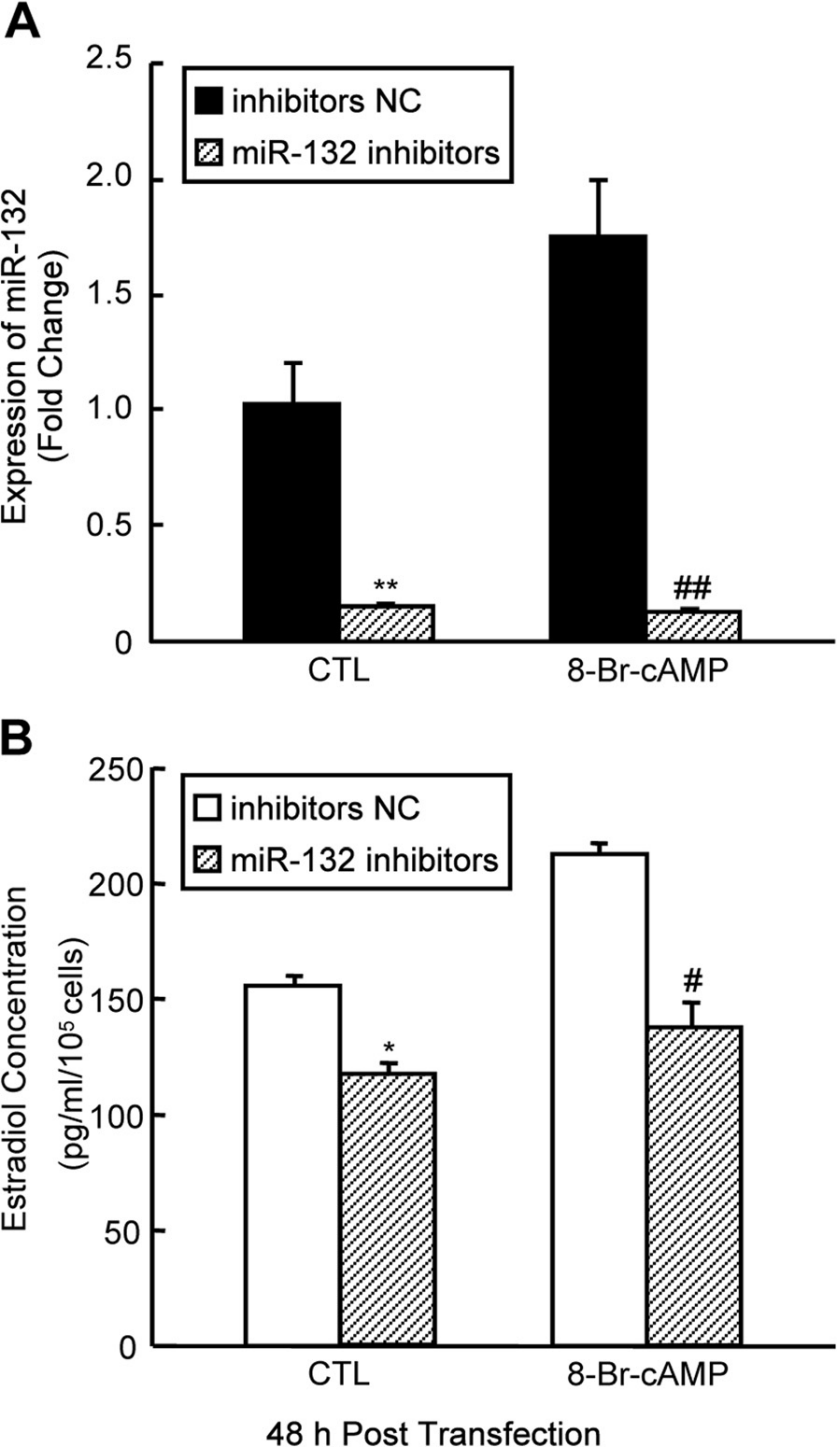
Downregulation of endogenous miR-132 inhibits E2 synthesis in mGCs. **a** mGCs were transfected with 100 nM miR-132 inhibitors or negative controls. Six hours after transfection, mGCs were cultured in the absence or presence of 8-Br-cAMP for another 48 h. The inhibition of endogenous miR-132 by specific inhibitors was validated by qRT-PCR. **b** The culture medium was collected for the measurement of E_2_ levels after endogenous miR-132 had been knocked down. The results represent the mean +/- SEM of three independent experiments performed in triplicate. *, # *p* < 0.05; **p < 0.01; ## *p* < 0.005, compared with the negative control (NC)

**Fig. 4:**
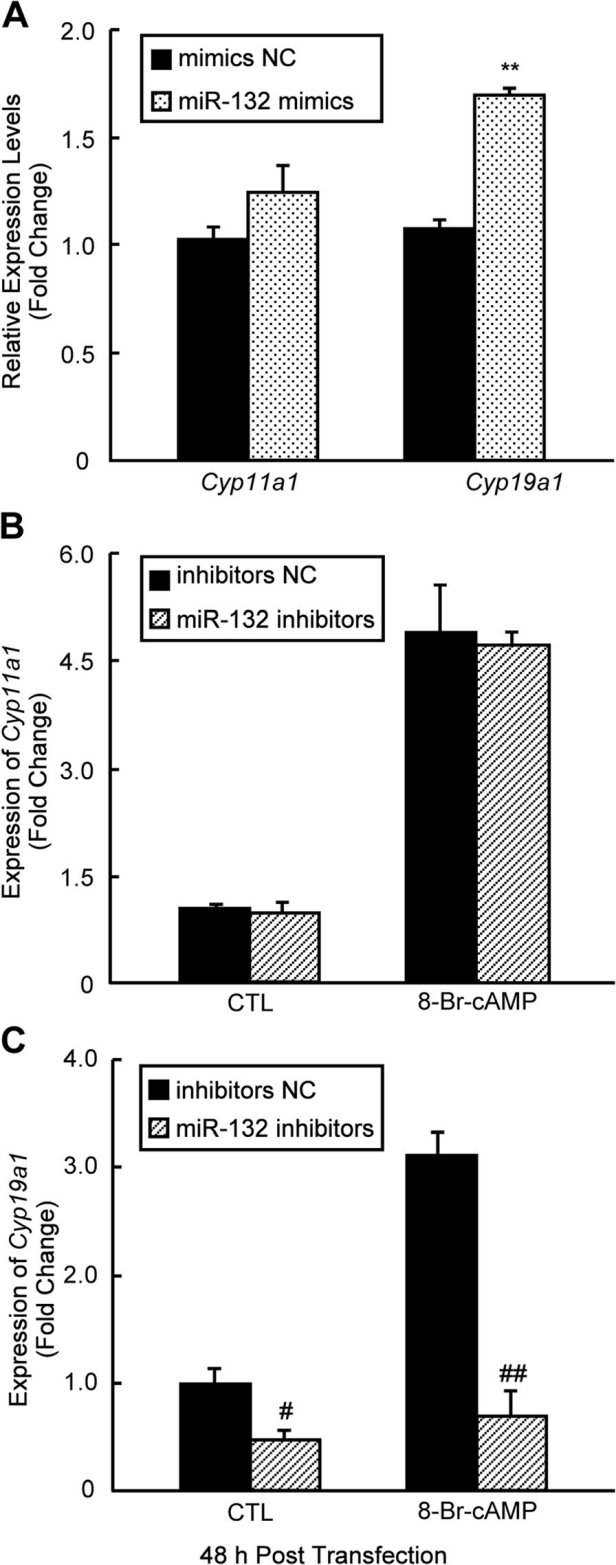
Effects of miR-132 on Cyp11a1 and Cyp19a1 in mGCs. To examine the effect of miR-132 on *Cyp11a1* and *Cyp191a1* transcription, mGCs were transfected with 100 nM of miR-132 mimics (**a**) or 100 nM of miR-132 inhibitors in the absence (**b**) or presence of 8-Br-cAMP (**c**) for 48 h as indicated, or the corresponding negative controls. Total RNA was extracted from mGCs 48 h after transfection, and the mRNA expression levels of *Cyp11a1* and *Cyp19a1* were measured by real-time PCR. The results represent the mean +/-SEM of three independent experiments. # *p* < 0.05; **p < 0.01; ## *p* < 0.005, compared with the negative control (NC)

Next, we assessed whether miR-132 has an effect on steroidogenesis in mGCs. To elevate miR-132 levels in mGCs, we transiently transfected mGCs with miR-132 mimics (i.e., chemically modified oligonucleotides) and confirmed the increased miR-132 levels using qRT-PCR (Fig. 2a). The progesterone levels changed only slightly (Fig. 2b). However, the E_2_ levels significantly increased after miR-132 overexpression. This increase was dose dependent; 35 % and 72 % increases in the E_2_ levels were observed when cells were transfected with 50 nM and 100 nM miR-132 mimics, respectively (Fig. 2c). In addition, we studied miR-132-related loss-of-function by knocking down endogenous miR-132 via the transient transfection of miR-132 inhibitors into mGCs (Fig. 3a). The results demonstrated that the synthesis of E_2_ was suppressed by miR-132 knockdown and downregulation of miR-132 prevented a cAMP-mediated increase of E_2_ in mGCs (Fig. 3b). The above findings suggest that miR-132 can serve as a stimulator for E_2_ synthesis in GCs. The detection of estrogen synthesis-related genes using real-time PCR revealed that a 1.6-fold increase in the expression of *Cyp19a1* (*P* < 0.01), the P450 aromatase gene required for E_2_ synthesis, was induced by miR-132 mimics (Fig. 4a). However, the expression of *Cyp11a1*, a key gene for progesterone synthesis, was not influenced by the overexpression of miR-132 (Fig. 4a). Significant effects on *Cyp11a1* expression were not observed after the knockdown of miR-132 in the presence or absence of 8-Br-cAMP treatment (Fig. 4b). In contrast, the observed effect of miR-132 inhibitors on *Cyp19a1* levels was similar to the suppression in E_2_ levels (Fig. 3b), and this effect became notable after 8-Br-cAMP treatment in mGCs (Fig. 4c). These findings suggest that miR-132 promotes E_2_ synthesis via the transcriptional regulation of aromatase but has little effect on progesterone synthesis due to its failure to regulate the transcription of *Cyp11a1*.

### miR-132 promotes *Cyp19a1* expression by downregulating *Nurr1*

**Fig. 5:**
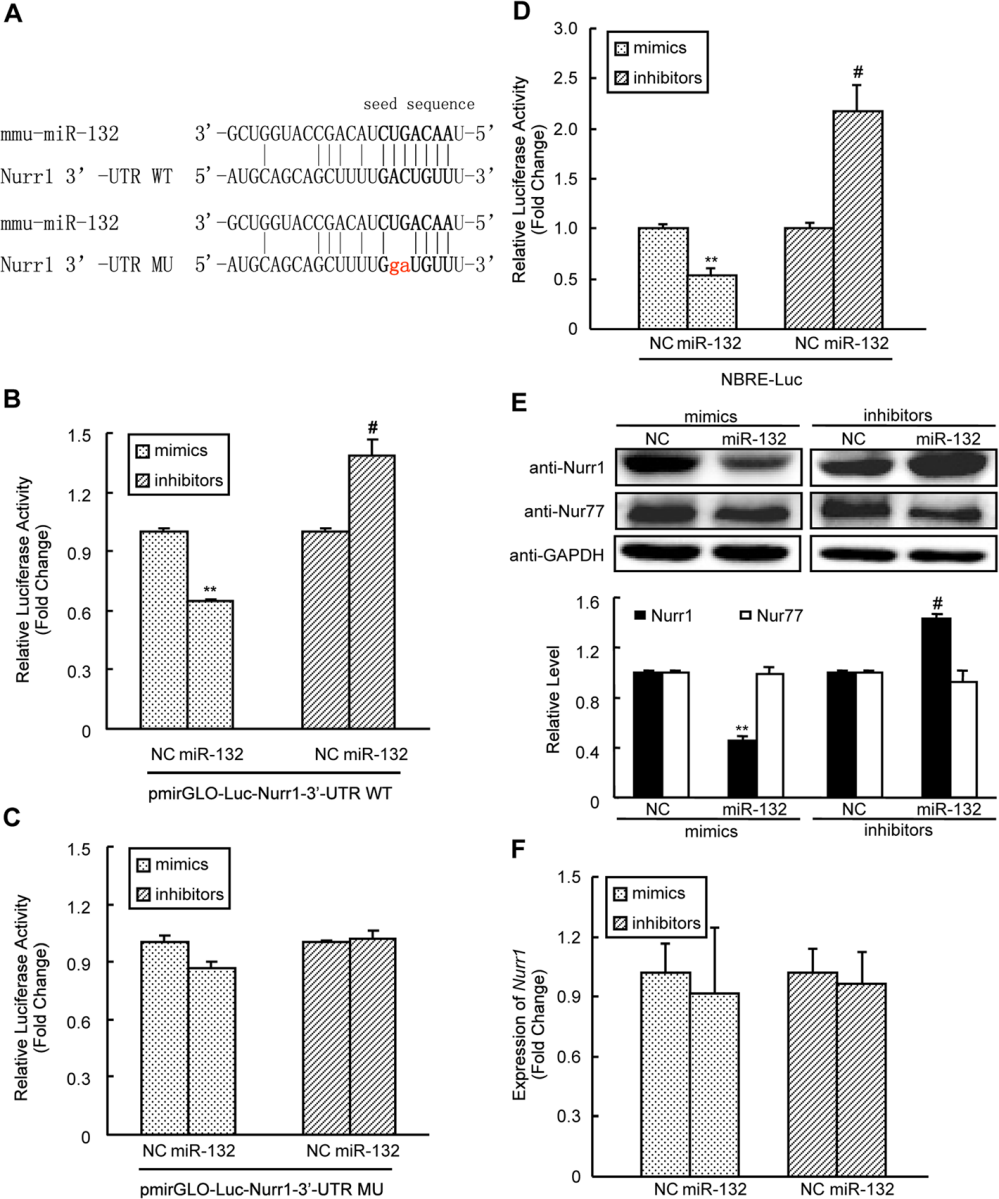
miR-132 suppresses Nurr1 expression post-transcriptionally. **a** The putative site in the Nurr1 3′-UTR that contains the ‘seed sequence’ for miR-132 binding. The g and a shown in red indicate the miR-132 binding site in the mutant form of Nurr1. mGCs were transfected with 100 nM miR-132 mimics, miR-132 inhibitors or the corresponding negative controls. Cells were co-transfected with the wild-type (WT) Nurr1 3′-UTR luciferase reporter plasmid (**b**), the mutant (MU) Nurr1 3′-UTR luciferase reporter plasmid (**c**) or the NBRE-luciferase reporter plasmid (**d**). After 48 h, luciferase assays were performed, and the results were normalized using constitutive Renilla luciferase. **e** Western blot analysis of Nurr1 and Nur77 protein expression in mGCs 48 h after transfection with 100 nM of miR-132 mimics, miR-132 inhibitors or the corresponding negative controls. The upper panels depict representative Western blots, and the lower panels present the statistical summary of the densitometric analysis from three independent experiments, indicating expression levels relative to negative controls after normalization to GAPDH. **f** Real-time PCR analysis of *Nurr1* mRNA levels in mGCs 48 h after transfection. The results represent the mean +/-SEM of three independent experiments performed in triplicate. **p < 0.01; # *p* < 0.05, compared with the negative control (NC)

miRNAs suppress translation by targeting the 3'-UTRs of mRNAs. We hypothesized that miR-132 promotes *Cyp19a1* expression by suppressing a *Cyp19a1* inhibitor. A bioinformatics screen using TargetScan (http://www.targetscan.org/vert_61/) revealed that the orphan nuclear receptor Nurr1, which suppresses aromatase expression via its PII promoter in KGN cells [29], is a putative target gene of miR-132. We constructed the luciferase reporter plasmid pmirGLO-Luc-Nurr1 3'-UTR WT, which contained the 3'-UTR of mouse *Nurr1* and the putative binding site for the ‘seed sequence’ of miR-132 (Fig. 5a), and co-transfected it into mGCs with either miR-132 mimics/inhibitors or the corresponding negative controls. Compared to controls, the overexpression of miR-132 significantly decreased luciferase activity, and the knockdown of miR-132 significantly increased luciferase activity in transfected mGCs (Fig. 5b), indicating that *Nurr1* is a direct target of miR-132. In addition, we constructed the pmirGLO-Luc-Nurr1 3'-UTR MU plasmid, which had two mutations in the ‘seed sequence’ of the miR-132 binding site, as indicated in Fig. 5a. miR-132 failed to affect the luciferase activity of the mutagenized Nurr1 3'-UTR plasmid (Fig. 5c). These results indicated that Nurr1 is a direct target gene of miR-132. The NR4A orphan nuclear factors bind as monomers to the NBRE motif (5'-AAAAGGTCA-3') and function in ligand-independent transcription activation. We utilized the NBRE-Luc luciferase plasmid, which contains tandem copies of the response elements for NR4A and drives a luciferase reporter gene, as a response reporter to reflect transcriptional activity of the NR4A family. The luciferase assay demonstrated that miR-132 suppressed the luciferase activity of the NBRE-Luc reporter gene. Our findings were further supported by the observation that the induction of the NR4A family was observed after the inhibition of endogenous miR-132 via miR-132 inhibitors (Fig. 5d). Analysis of Nurr1 levels via Western blot analysis demonstrated that Nurr1 protein expression was significantly lower in mGCs that were transfected with miR-132 mimics than in mGCs that were transfected with mimics NC. In contrast, compared to treatment with inhibitors NC, knockdown of miR-132 using miR-132 inhibitors led to increased Nurr1 protein expression in mGCs (Fig. 5e). These results were consistent with the findings of a previous study of the differentiation of dopamine neurons [30]. Significant changes in *Nurr1* mRNA levels were not observed after either the overexpression or knockdown of miR-132 (Fig. 5f). In the 3'-UTR of *Nur77*, which has also been identified as a repressor of aromatase in NR4A family members, no putative binding site for miR-132 was found via bioinformatics screening. The expression level of the Nur77 protein was not influenced by miR-132 (Fig. 5e) in our study. In summary, miR-132 post-transcriptionally inhibits the translation of Nurr1 and weakens its repressive effect on *Cyp19a1* transcription. This miR-132-mediated reduction of Nurr1 repression leads to *Cyp19a1* upregulation and increased E_2_ synthesis.

### Knockdown of *Nurr1* partially attenuates the effects of miR-132 and re-expression of *Nurr1* abrogates the stimulatory effect of miR-132 on E_2_ synthesis

**Fig. 6:**
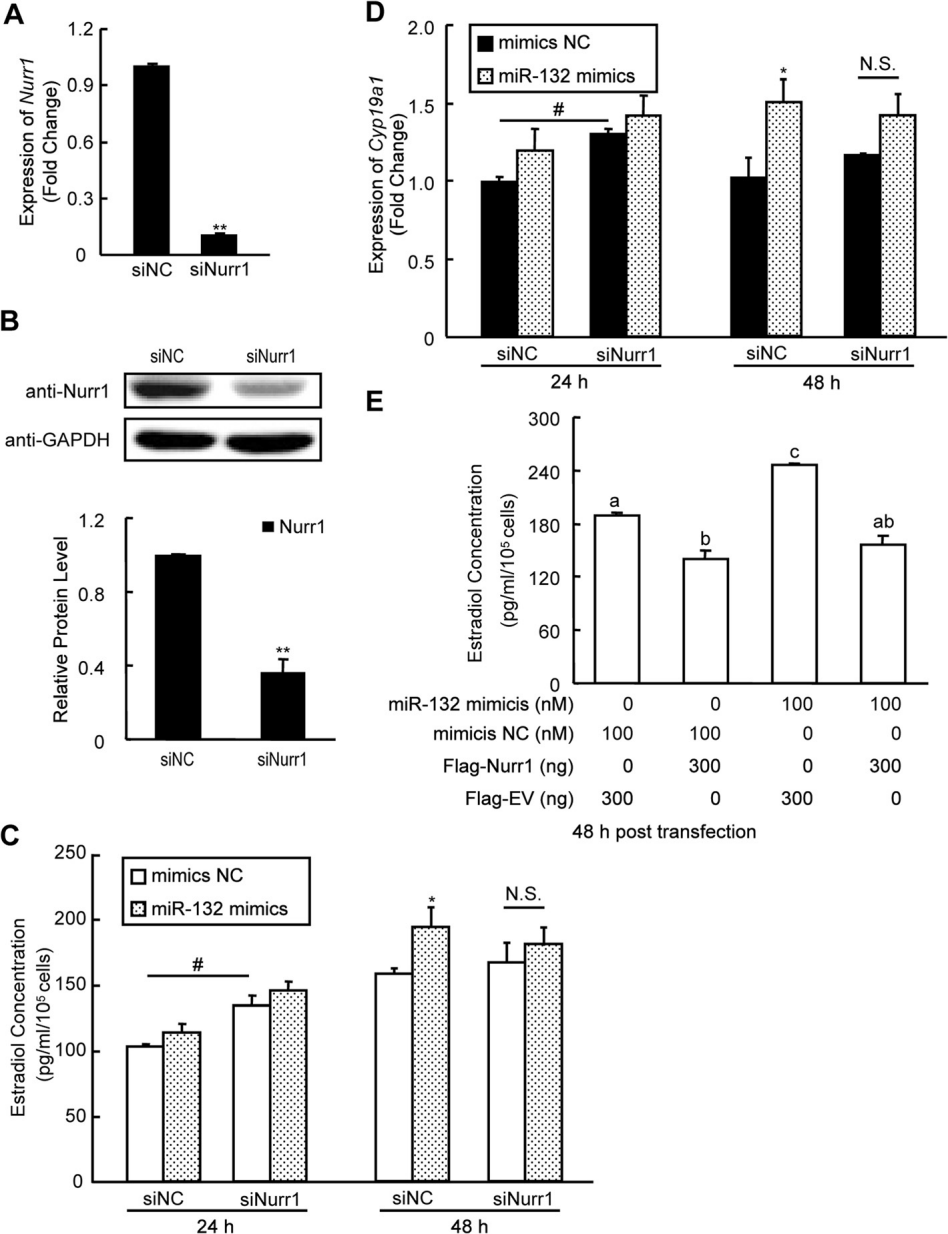
Knockdown of Nurr1 partially attenuates the effects of miR-132, and re-expression of Nurr1 abrogates the stimulatory effect of miR-132 on E2 synthesis. **a** mGCs were transfected with 100 nM siRNA targeting *Nurr1* or negative control siRNA. Real-time PCR detection of *Nurr1* mRNA levels was performed 48 h after transfection of mGCs. **b** After knockdown, Nurr1 protein levels were analyzed by Western blot. **c** siRNA was transfected 24 h before transfection of miR-132 mimics or negative controls, followed by continuous culture for an additional 48 h. The culture medium was collected for the measurement of E_2_ levels 24 h and 48 h after transfection. **d** Total RNA was extracted from mGCs and *Cyp19a1* mRNA levels were measured using real-time PCR. **e** Culture medium was collected for the measurement of E_2_ levels 48 h after transfecting mGCs with the Flag-Nurr1 or the Flag-empty vector (EV) plasmids and miRNA mimics, as indicated. The results represent the mean +/-SEM of three independent experiments performed in triplicate. Values with different superscripts (a, b, c) are significantly different (*p* < 0.05). *p < 0.05; **p < 0.01; N.S. no significant difference, compared with the negative control (NC)

The knockdown of *Nurr1* via RNA interference was validated using both Western blot analysis and real-time PCR (Fig. 6a, b). The E_2_ synthesis was primarily elevated after *Nurr1* knockdown via siNurr1 transfection compared to siNC (Fig. 6c, the left panels), which was similar to the previously observed effect of the downregulation of *Nurr1* by miR-132. Followed by transfection of miR-132 mimics for 48 h, miR-132 significantly promoted E_2_ synthesis as expected in siNC group, while miR-132 failed to further contribute to the elevation of the E_2_ synthesis in siNurr1 group (Fig. 6c, the right panels). In addition, the *Cyp19a1* mRNA levels showed similar changes (Fig. 6d). After transfecting mGCs with Flag-Nurr1, which upregulated Nurr1 protein levels independent of miR-132 repression due to the absence of the 3'-UTR binding sequence for miR-132 in the pFLAG-CMV-2 expression plasmid, the stimulatory effect of miR-132 on E_2_ synthesis was largely abrogated (Fig. 6e). The E_2_ levels dropped to basal levels. These results suggest that Nurr1 plays an important role in miR-132-induced E_2_ synthesis.

## Discussion

Both FSH and LH promote intracellular cAMP in GCs via binding to their receptors [23]. We isolated naïve GCs from immature mice and used 8-Br-cAMP to mimic the secondary messenger downstream of the FSH pathway. Our *in vitro* analysis of miR-132 expression in cultured mGCs treated with 8-Br-cAMP demonstrated that miR-132 levels were significantly upregulated and peaked at 12 h (Fig. 1e). The induction of miR-132 was also observed in FSH or cAMP -treated rat GCs [19, 20]. The treatment of mouse ovaries with an ovulatory dose of LH/hCG revealed that miR-132 was highly upregulated in periovulatory mGCs [21]. The results of previous studies are consistent with our findings, which demonstrated that miR-132 was induced by hormonal stimulation and activation of the cAMP pathway in GCs. A previous study demonstrated that miR-132 is regulated by CREB via CRE motifs upstream of miR-132 [31]; this finding explains the observed upregulation of miR-132 by cAMP activation in GCs. These findings also suggest that miR-132 mediates functions of the cAMP pathway during the differentiation process of GCs.

There is increasing interest in identifying the functions of miRNAs in GCs. miR-21, which in addition to miR-132 and miR-212, is an LH-induced miRNA, blocks apoptosis in mGCs [32]. The TGF-β/Smads signaling pathway plays critical roles in early follicle development, GC proliferation and differentiation. Our previous study demonstrated that miR-145 and miR-181a suppress the proliferation of mGCs by targeting Acvr1b and Acvr2a, respectively [25, 33]. This pathway also regulates the expression of many miRNAs, including miR-224 and miR-383 [15, 16]. Elevated miR-224 can enhance TGF-β1-induced mGC proliferation by targeting Smad4 and ovarian E_2_ release [15], while the downregulation of miR-383 promotes steroidogenesis by targeting *RBMS1* and can be transactivated by SF-1 through direct binding to the promoter of the miR-383 host gene *SGCZ* [16]. In porcine GCs, miR-378 is spatiotemporally expressed and shows an inverse expression pattern to that of aromatase. Aromatase expression and subsequent E_2_ production by GCs are directly post-transcriptionally downregulated by miR-378 [19]. In KGN cells, overexpression of miR-132 increased E_2_ levels [22], which is consistent with our findings in mGCs. However, a study in equine follicle development found that miR-132 was increased in granulose cells from luteinizing follicles with higher progesterone and lower estradiol concentration in the follicular fluid [34]. In preovulatory mGCs, knockdown of miR-132 failed to affect estradiol or progesterone after cAMP treatment [21]. In a genome-scale screen of steroid hormone release influenced by miRNAs in human primary ovarian GCs, 51 miRNAs were found to suppress E_2_ release, whereas none of the miRNAs (including miR-132) studied were found to have a stimulatory effect on the E_2_ level [14]. This discrepancy could be attributed to differences between species and cell models. miR-132 may exhibit diverse functions at specific stages of GCs development. Therefore, we utilized a lower plating density to retain an estrogenic phenotype of GCs. Our data suggest that E_2_ production and the *Cyp19a1* mRNA levels in mGCs are elevated by miR-132 directly. Our loss-of-function study also demonstrated that the knockdown of miR-132 could downregulate the expression of *Cyp19a1*. Consequently, the increased levels of miR-132 after 8-Br-cAMP treatment could contribute to the extended suppressive effect of miR-132 inhibitors on *Cyp19a1*. Taken together, miR-132 was induced by cAMP and likely mediated the FSH pathway in the primary cultured mGCs that we studied because of its stimulatory effect on E_2_ synthesis. To better understand the functions of miR-132 in GCs of terminal differentiation (e.g. apoptosis), further studies are needed.

In addition, our research elucidated some of the molecular mechanisms that underlie the stimulatory effect of miR-132 on E_2_ synthesis. We hypothesized that miR-132 stimulates E_2_ synthesis via translational regulation of an orphan nuclear receptor*-Nurr1*. Orphan nuclear receptors in the ovary, such as SF-1, which is also known as NR5A1 [6, 35], are emerging as important ovarian factors that regulate female reproduction. The orphan nuclear receptor Nurr1 belongs to the nuclear receptor subfamily 4A (NR4A) subgroup along with Nur77 and Nor1 [29]. The genes encoding these transcription factors are classified as immediate early response genes because their expression is rapidly induced by a variety of physiological stimuli, including fatty acids, prostaglandins, growth factors, calcium, cytokines and peptide hormones (e.g., FSH) [36]. NUR77 is a novel transcription factor that contributes to the regulation of prolactin gene expression in human endometrial stromal cells and regulates androgen receptor gene expression in ovarian GCs [37, 38]. Both NUR77 and NURR1 suppress the transcription of aromatase and modulate its expression in the KGN human granulosa-like tumor cell line [29]. In a recent study of embryonic stem cell differentiation, miR-132 was demonstrated to directly regulate the expression of *Nurr1*, which is an important transcription factor in dopamine neuron development and differentiation [29]. Our study demonstrates that miR-132 suppressed Nurr1 expression by targeting its 3'-UTR (Fig. 5b). Interestingly, the Nurr1 protein levels in mGCs were dramatically decreased by the overexpression of miR-132 (Fig. 5e), whereas the *Nurr1* mRNA levels were only slightly changed (Fig. 5f). This finding indicates that in mGCs, miR-132 induces *Nurr1* translation inhibition but not mRNA degradation by binding to the 3'-UTR of *Nurr1.* It has been suggested that the promoter-proximal region of the aromatase PII promoter, which contains the binding sites for SF-1 and a CLS, also mediates the transcriptional repression of NURR1 and NUR77 [29]. However, this protein-DNA interaction might be too transient or too weak to be detected by the gel shift assay used in the previous study. The underlying mechanism by which NR4A mediates the transcriptional repression of *Cyp19a1* remains to be elucidated. In contrast to the previously reported transient peaks in NR4A expression, the cAMP-mediated induction of miR-132 resulted in a delayed elevation pattern [29]. Conceivably, miR-132 expression could contribute to the decline of *Nurr1* and the subsequent upregulation of *Cyp19a1*.

A previous study demonstrated that in neurons, miR-132 is regulated by multiple factors, such as BDNF [39], and is required for both neuronal morphogenesis and long-term synapse activation [28]. Some targets of miR-132, including *p250GAP* [40] and *MeCP2* [41], have been identified. Interest in the involvement of miR-132 in endocrine biology has emerged recently. miRNA profiling in LβT2 cells exposed to gonadotropin-releasing hormone revealed the significant induction of miR-132, which subsequently regulated cellular motility [42]. Our study suggests that miR-132 may exert differential effects on reproductive endocrine regulation (e.g., the promotion of estrogen synthesis). In light of the important roles of both miR-132 and estrogen in brain function, it would be of interest to determine whether miR-132 influences local estrogen synthesis in the nervous system. In addition, the induction of miR-132 during Kaposi’s sarcoma-associated herpes virus infection represses the expression of p300, a co-activator of CREB, which acts as part of a negative feedback loop that leads to the inhibition of miR-132 expression and the restoration of p300 expression [43]. This regulatory network may contribute to the observed decline in miR-132 levels after peak expression is reached during cAMP treatment. The precise regulatory role of miR-132 and its functions in GCs remain to be elucidated. In addition, further *in vivo* studies, such as a study using floxed miR-212/132 mice [44] to specifically ablate miR-132 in GCs, could improve our understanding of the effect of miR-132 on E_2_ synthesis. A recent study in polycystic ovary syndrome patients found that the expression levels of miRNA-132 in follicular fluid were significantly lower in patients than in controls [22]. The dysfunctions of miR-132 in the development of polycystic ovary syndrome and premature ovarian failure are to be elucidated in future studies.

## Conclusions

In summary, our study demonstrated that cAMP induces the expression of miR-132 in mGCs; E_2_ synthesis is subsequently induced by miR-132 via the upregulation of *Cyp19a1*. miR-132 induces *Cyp19a1* by directly suppressing the expression of *Nurr1*. The observed effects of miR-132 on physiological processes in GCs may be useful for regulating reproduction and treating steroid-related disorders.

## Abbreviations

3'-UTR: 3'-untranslated region
cAMP: Cyclic adenosine monophosphate
E_2_: Estradiol
GCs: Granulosa cells
miR-132: microRNA-132
